# Post-stroke fatigue: an exploratory study with patients and health professionals to develop a patient-reported outcome measure

**DOI:** 10.1186/s41687-021-00307-z

**Published:** 2021-04-21

**Authors:** Ingrid Johansen Skogestad, Marit Kirkevold, Petra Larsson, Christine Råheim Borge, Bent Indredavik, Caryl L. Gay, Anners Lerdal

**Affiliations:** 1 Medical Department, Lovisenberg Diaconal Hospital, Oslo, Norway; 2 Department of Nursing Science, Faculty of Medicine, Institute of Health and Society, University of Oslo, Oslo, Norway; 3 Department of Nursing and Health Promotion, Oslo Metropolitan University, Oslo, Norway; 4 Surgical Department, Lovisenberg Diaconal Hospital, Oslo, Norway; 5 Department for Interdisciplinary Health Sciences, Faculty of Medicine, Institute of Health and Society, University of Oslo, Oslo, Norway; 6 Department of Research and Development, Lovisenberg Diaconal Hospital, Oslo, Norway; 7 Department of Neuromedicine and Movement Science, NTNU, Trondheim, Norway; 8 Department of Stroke, St. Olavs Hospital, Trondheim, Norway; 9 Department of Family Health Care Nursing, University of California San Francisco, San Francisco, USA

**Keywords:** Fatigue, Stroke, Rehabilitation, Qualitative research, Patient-reported outcome measure

## Abstract

**Background:**

Post-stroke fatigue (PSF) is commonly reported and described as disabling by patients recovering from stroke. However, a major challenge is how to accurately diagnose and assess PSF. Therefore, the aim of this study was to explore PSF as it is experienced by stroke survivors and described by health professionals to guide future development of a PSF-specific PROM.

**Methods:**

Individual semi-structured interviews were conducted with stroke survivors experiencing PSF (*n* = 9) and three focus groups were conducted with health professionals (*n* = 16). Data were analyzed through inductive content analysis.

**Results:**

The analysis revealed four themes illustrating the experience and descriptions of PSF: 1) PSF characteristics, 2) interfering and aggravating factors, 3) management, and 4) PSF awareness, which refers to stroke survivors first becoming aware of PSF after their initial hospital admission.

**Conclusion:**

This study highlights the complexity and multidimensionality of PSF. The results from this study will guide future development of a PSF-PROM and support its content validity.

**Supplementary Information:**

The online version contains supplementary material available at 10.1186/s41687-021-00307-z.

## Introduction

Post-stroke fatigue (PSF) is one of the most common symptoms 3 months after stroke [1] and can have negative implications for patients’ rehabilitation, physical function, activities in daily life, and quality of life [2–4]. Despite the high prevalence (25–85%) [5] and disabling nature, evidence-based interventions to prevent and treat PSF are currently lacking [6]. A major challenge is to achieve accurate diagnostics of PSF, a prerequisite for the development of novel preventive and therapeutic measures [7].

PSF can be defined as lack of energy, or increased need to rest, every day or nearly every day, leading to difficulties partaking in everyday activities [8]. The diagnosis of PSF is traditionally based on patient-reported outcome measures (PROMs). However, a recent review showed that the PROMs most commonly used to measure fatigue in stroke survivors have several limitations [9]. This is in line with a previous review, which did not find any fatigue PROM that met critical criteria for an ideal instrument [10]. Moreover, existing fatigue PROMs mostly include only one or two fatigue dimensions, such as the intensity or impact of fatigue, and do not assess other potentially relevant dimensions of the fatigue experience [9]. In addition, existing fatigue PROMs are not developed specifically for stroke survivors [11]. The former shortcomings of these fatigue PROMs may partly be explained by the lack of involvement of patients and health professionals in the instrument development process [9], which is strongly recommended in guidelines [12, 13] and often a part of the health technology assessment for medicinal product approval and reimbursement [14, 15].

Although relatively few qualitative studies have been published on PSF, they consistently describe fatigue as having several dimensions [16]. This includes core characteristics of PSF [16], different factors contributing to fatigue [17, 18], and various aspects of daily life affected by fatigue [19]. However, previous qualitative studies have not aimed to guide item development in a new PSF-specific PROM. Qualitative studies, through interviews or focus groups, are the preferred method to establish content validity in new PROMs [20]. Qualitative studies have the advantage of being able to directly engage with the experts, who can provide a comprehensive understanding of the construct to be measured. Experts include both patients and health professionals. Patients have the symptom experience, and health professionals have clinical experience treating these patients and can describe typical characteristics, consequences and management of the symptom [13, 20–24]. A qualitative study will provide a deeper understanding of PSF, how it impacts life, and management strategies, which is critical to addressing the limitations of existing fatigue PROMs and informing the content and structure of a PROM specifically developed to measure PSF.

The overall aim of this study was to explore PSF as it is experienced by stroke survivors and described by health professionals to guide future development of a new PSF-specific PROM.

## Methods

### Design

In this qualitative study, individual semi-structured interviews were conducted with stroke survivors with PSF and multi-disciplinary focus groups with health professionals who provide clinical care to stroke patients. This study was conducted in Norway as part of a larger research project, which includes three sub-studies with the overall aim of developing and testing a new PROM for PSF. This study has followed COSMIN criteria for establishing content validity in PROMs [13], as well as the COREQ checklist for qualitative studies (Online resource 1) [25].

### Participants

#### Stroke survivors

Nine stroke survivors were included in this study. The inclusion criteria were: (1) stroke within the last 2 years, (2) 18 years or older, and (3) meeting the diagnostic criteria for PSF as defined by a clinical interview [8]. For the first criterion, all types of stroke were included, as defined by the International Classification of Diseases 10th edition (ICD-10) [26] and included codes for ischemic stroke (I63), non-traumatic intracerebral haemorrhage (I61), and stroke, not specified as haemorrhage or infarction (I64). The time period of stroke within the last 2 years was chosen because the level of post-stroke fatigue has previously been shown to remain constant for up to 2 years [27]. A purposive sampling strategy was used and aimed to recruit participants with diversity in age, gender, physical impairment, communication disorders and living accommodations. As these demographic and clinical variables may influence patients’ PSF experiences, such a purposive sampling strategy was intended to provide different perspectives and descriptions of PSF [23, 24]. Five stroke participants were recruited through a Facebook page for a stroke user organization, and four participants were recruited from the stroke outpatient clinic at a hospital in Oslo, Norway. Additional information is provided in the COREQ checklist including sampling strategy, study design, analysis and research team (Online resource 1).

#### Heath professionals

A total of 16 health professionals participated in 3 focus groups. All health professionals were involved in the clinical care of stroke patients, and recruited from different levels of health care services and from different disciplines. Participants with varied ages, genders, professions and years of clinical experience were recruited to obtain diverse perspectives on PSF.

### Data collection

The first author conducted all the interviews and focus groups, and a co-author co-moderated the focus groups, observing and taking notes. Based on the study’s aim and previous literature reviews [2, 16], an interview guide was developed to ask: 1) how PSF is described from the perspective of stroke survivors, 2) what it is like to live with PSF, from the perspectives of both stroke survivors (based on personal experience) and health professionals (based on clinical observation), and 3) how PSF is managed by stroke survivors and by health professionals (Online resource 2).

Most interviews and focus groups lasted 60–80 min, while one interview lasted 45 min. All interviews and focus groups were audio-recorded and transcribed verbatim by the first author. Reflection notes were made immediately after completing each interview/focus group, which aimed to describe contextual information as well as immediate reflections on the data. In addition to interviews, we obtained the following data from the stroke survivors: demographics, stroke characteristics and clinical outcome, modified Rankin Scale (MRS) [28], fatigue (Fatigue Severity Scale [FSS]) [29], depression and anxiety (Hospital Anxiety and Depression Scale [HADS]) [30, 31], cognitive function (Montreal Cognitive Assessment [MOCA]) [32], and health-related quality of life (EuroQol five dimension scale [EQ-5D]) [33, 34]. Focus group participants answered questionnaires about their age, gender, profession, and years of experience with stroke.

### Data analysis

Methods for inductive content analysis were applied with the aim to identify, analyse and report themes and categories of stroke survivors’ experiences and health professionals’ descriptions of PSF [35, 36]. The analysis included reading, open coding, organizing and abstracting codes into sub-categories, categories and themes, and reporting the results. Data collection and analysis occurred simultaneously as an iterative process. The decision to stop data collection was based on a comprehensive evaluation including considerations of the study aim, interview quality and when analysis revealed no new categories in the additional data [35, 37].

Upon completion of each interview, transcripts were imported to NVIVO (v.11), a qualitative data analysis software used to enhance efficiency and transparency in the analytical process [38]. Analysis of the two populations was done separately and was combined for reporting the results. The transcripts were read and re-read several times to get a sense of the whole material. The coding process started after conducting the first three interviews and continued consecutively throughout the process of data collection and analysis. First, the material was coded by its manifest content, i.e. the level of interpretation and abstraction was low at this point. The analytical units in each transcript were given one or more individual codes. All codes were grouped according to their content and formed sub-categories and categories. These categories were first formed separately within each transcript, before further analyses of these categories across the data material in each of the two populations. This method allowed for transparency in the process of finding major and minor categories, patterns between categories, and similarities and differences across the stroke survivors and health professionals. All of the sub-categories were abstracted to categories, which further represented four themes. Examples are presented in Table 1.

**Table 1:** Analysis, examples of themes, categories, sub-categories and codes

Theme	Categories	Sub-categories	Code
Characteristics	Quality	Mental fatigue	Mentally exhausted
			Not physically tired
			Head feels heavy
Interference and aggravating factors	Cognitive performance		Communication difficulties when fatigued
			Concentration difficulties when fatigued
			Fatigued by decision-making
			Prolonged attention induces fatigue

Questionnaire data were summarized using descriptive statistics (i.e., frequencies, medians, ranges) to describe the characteristics of participating stroke survivors and health professionals.

### Ethical considerations

The study was approved by the Regional Medical and Health Ethics Committee of Southeastern Norway (REK), with reference [reference removed due to blinding]. All participants received written information about the study, gave written consent and were informed about their ability to withdraw from the study at any time before publication of results.

## Results

**Fig. 1:**
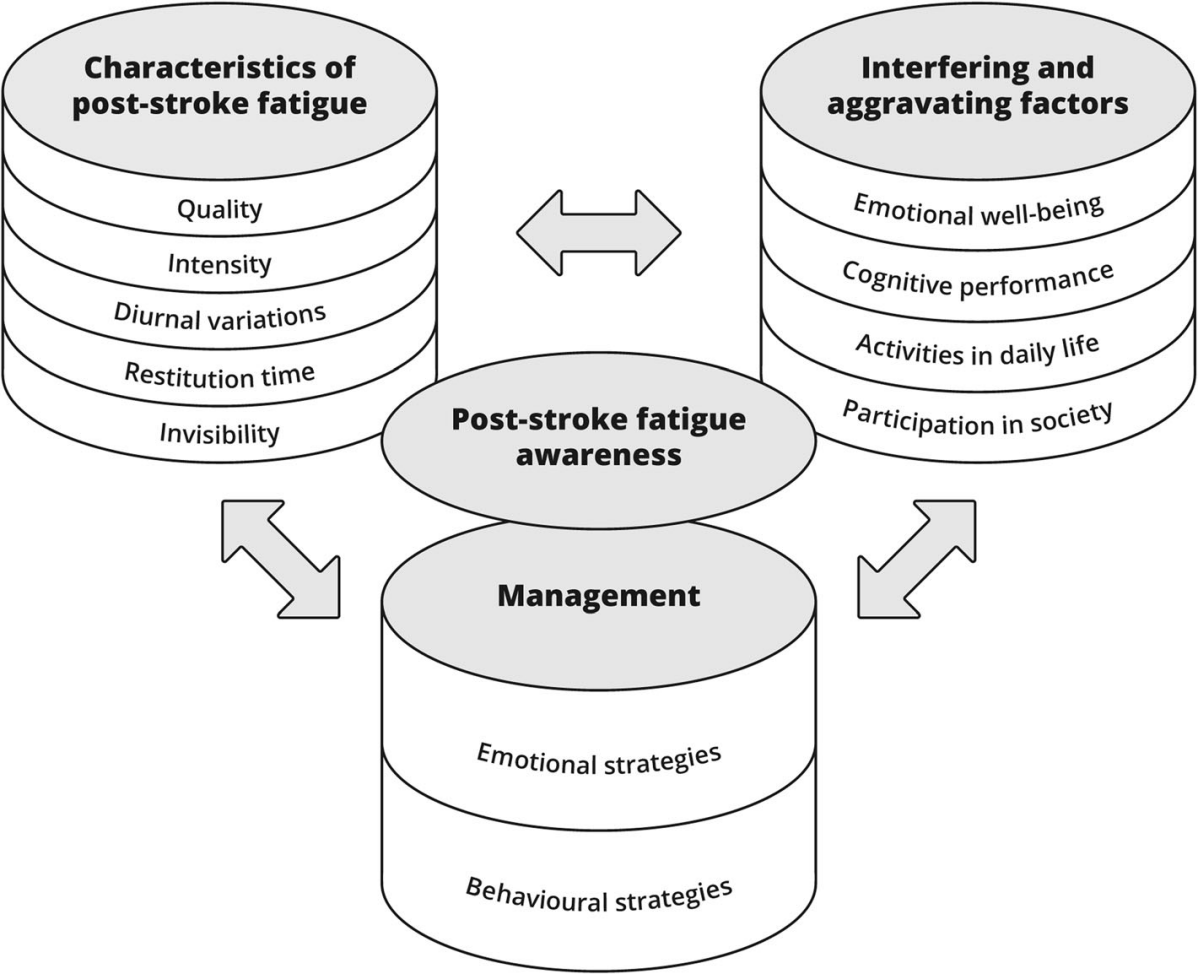
Conceptual model of post-stroke fatigue, including themes and categories. PSF has different characteristics that affected how PSF interfered with patients’ lives. These interfering and aggravating factors had to be managed, and the use of management strategies could again influence the characteristics of PSF and whether or how PSF continued to interfere with their lives. Fatigue was initially interpreted as a normal reaction to having a stroke, and stroke survivors first became aware of PSF sometime after their initial hospital admission

Analysis of the data material revealed four themes that illustrate the experiences and descriptions of PSF: characteristics, interference and aggravating factors, management, and PSF awareness (Fig. 1). Results from the individual interviews with stroke survivors and from the focus groups with health professionals were mostly consistent, and focus group findings are only reported when they contributed additional information. Characteristics of the 9 participating stroke survivors are summarized in Table 2 and characteristics of the 16 participating health professionals are summarized in Table 3.

**Table 2:** Demographics, clinical characteristics and health-related quality of life of the stroke survivors with PSF (individual interview participants)

Stroke Survivor Characteristics	n	Median (range)
**Time since stroke, in months**		21 (3–24)
3–8 months	4	
20–24 months	5	
**Age in years**^a^	9	59 (23–80)
**Gender**		
Male (female)	4 (5)	
**Living arrangements**		
Living with a partner	6	
Living with children or other family member	2	
Living alone	1	
**Residence**		
Urban area	5	
Rural area	4	
**Education**		
Upper secondary education	3	
Higher education < 4 years	3	
Higher education ≥ 4 years	3	
**Work status**		
*Pre-stroke*		
Full time work/studies (100%)	5	
Retired	4	
*Post-stroke*		
Disability leave (100%)	2	
Partial sick leave (50% – 70%)	3	
Retired	4	
**ICD-10 Classification**^b^		
Non-traumatic intracerebral hemorrhage (I61)	3	
Cerebral infarction (I63)	6	
**Degree of disability at stroke onset (mRS)**^c^		
Moderate severe disability (mRS 4)	1	
Moderate disability (mRS 3)	1	
Slight disability (mRS 2)	5	
No significant disability (mRS 1)	2	
**Communication disorder at stroke onset**^d^		
Aphasia (self-reported)	4	
Normal speech	5	
**Living situation** (at the time of the interview)		
Dependent living (assistance provided)	1	
Independent living (no assistance provided)	8	
**Rehabilitation services** (at the time of the interview)^e^		
Physiotherapy (weekly)	3	
None	6	
**Fatigue (FSS7)**^f^		6.4 (4.7–7)
Severe fatigue (FSS ≥ 5)	7	
Moderate fatigue (FSS = 4–4.9)	2	
**Depression and anxiety (HADS total score)**^g^		20 (9–23)
Likely case of depression and/or anxiety (HADS ≥19)	5	
Possible case of depression and/or anxiety (HADS 15–18)	2	
Normal symptoms of depression and anxiety (HADS < 15)	2	
**MoCA**^h^		26 (22–29)
Mild cognitive impairment (MoCA 18–25)	4	
No cognitive impairment (MoCA ≥26)	5	
**Self-reported health EQ-VAS**^i^	9	40 (30–80)

^a^ The individual ages of the participants were: *23-54 - 54 - 55 - 59 - 74 - 76 - 79 - 80*

^b^ The ICD-10 classification is based on the participants retrospective self-report (*n* = 5) or collected from their medical record (*n* = 4)

^c^ *MRS* Modified Rankin Scale measures the degree of stroke impairment or dependence in daily activities. Scores can range from no symptoms (0) to death (6). MRS in this study was rated by IJS, based on retrospective self-report (*n* = 5) or medical records (*n* = 4) [28]

^d^ Communication disorder at stroke onset was based on self-report. None of the participants had significant aphasia at the time of the interview

^e^ None of the participants received any other rehabilitation services at the time of the interview such as occupational therapy, speech-language therapy etc.

^f^ *FSS7* Fatigue Severity Scale 7-item version is scored on a 7-point scale ranging from strongly disagree (1) to strongly agree (7). An individual mean score of ≥5 indicates severe fatigue, score 4–4.9 indicates moderate fatigue, and score < 4 indicates no/mild fatigue [29]

^g^ *HADS* Hospital Anxiety and Depression Scale is a screening instrument developed to identify depression and anxiety in medical patients. HADS total score ≥ 19 indicates a case of depression and anxiety, a score between 15 and 18 indicates a possible case, and scores below 15 indicates no symptoms of depression or anxiety [30, 31]

^h^ *MoCA* Montreal Cognitive Assessment is a screening instrument developed to detect mild cognitive impairment. Scores over 25 indicate normal cognition and scores between 18 and 25 indicate mild cognitive impairment [32]. In stroke patients, normal scores range 20–27 during the chronic post-stroke phase [39]

^i^ EQ-VAS assesses overall health-related quality of life, ranging from *worst possible* (0) to *best possible* (100) health [33]. Mean EQ-VAS score in a Norwegian general population sample is 77.9 (SD = 19.5) [34]

**Table 3:** Background characteristics of health professionals (focus group participants)

Health Professional Characteristics	Median (range)
**Age**	48.5 (26–58)
**Years of experience with stroke patients**	15.5 (3–32)
	**n**
**Gender**	
Male (female)	7 (9)
**Place of work**	
Stroke unit at a local hospital	6
Stroke rehabilitation hospital	5
Community home care	5
**Profession**	
Physiotherapist	5
Occupational therapist	5
Nurse	3
Speech therapist, physician, clinical psychologist	3

### Characteristics of PSF

In the data material from both stroke survivors and health professionals, PSF was described as a complex and multifactorial phenomenon. For the theme *characteristics of PSF* five categories were identified: *quality, intensity, diurnal variations, restitution time, and invisibility* (Fig. 1)*.*

Perceptions of PSF varied between the stroke survivors, and different descriptions of fatigue *quality* included three sub-categories: mental fatigue, physical fatigue, and general fatigue. Some described feeling mentally fatigued, whereas for others, fatigue presented itself physically as a bodily sensation. However, most used general terms, such as:

*“I need to rest my head, I get exhausted in my head, and then I also become tired in my body.”* (Participant 4)

*“I have not done anything other than just sit still [ … ] It is not really tiredness either, it is just a completely different experience, not tired and not sleepy, but a combination of those two, but in a COMPLETELY different way than before the stroke.”* (Participant 7)

The *intensity* of fatigue spanned from total exhaustion that prevented the stroke survivors from completing ordinary duties, to manageable fatigue. The stroke survivors also experienced *diurnal variations* of fatigue. Most had days or times during a day without fatigue, and some of them described waking up refreshed, whereas others were fatigued in the morning. The stroke survivors described different patterns of diurnal variations, but a common feature was that the levels of fatigue varied throughout the day. The stroke survivors could also have days or weeks of feeling better or worse. These diurnal and periodic variations of fatigue were a prominent characterization of their experiences.

*“It [fatigue] is bad in the morning, but then it gets better. It is usually best at mid-day, and then early afternoon it is a dead break [ … ] and in the evening … every evening it is just as if I have used all the energy [ … ] then I am very tired...”* (Participant 7)

When the stroke survivors experienced fatigue, they needed long *restitution time*. The actual recovery time needed varied, but in general, longer restitution time was needed after the stroke. They further described how PSF was *invisible* and that other people had difficulties recognizing their fatigue:

*“It is my level of energy … the invisible complaints that are a challenge [ … ] I look quite well, but I have been sick and I am still marked by that … ”* (Participant 3)

The stroke survivors also had difficulties finding appropriate words to describe PSF to others and to explain how their fatigue was distinct from regular tiredness. This invisibility of PSF constituted the final category of PSF characteristics.

### Interfering and aggravating factors

The stroke survivors described how fatigue *interfered* with their lives and how different factors *aggravated* fatigue. Four categories of interfering and aggravating factors were identified: *emotions, cognitive performance, activities in daily life, and participation in society.* Having fatigue interfered with all these aspects of their lives, and in addition, different factors in all four categories could aggravate their fatigue.

PSF also affected the stroke survivors’ *emotions*. Having an acute stroke was a frightening experience for the stroke survivors, and the continuous presence of fatigue after the stroke perpetuated their perception of feeling unwell. This contradicted the stroke survivors’ understanding of their stroke as a one-time incident from which they had fully or mostly recovered. The stroke survivors also lacked motivation, worried that people would perceive them as lazy and experienced sadness related to how fatigue interfered with their lives:

*“I have to say that it is quite depressing. Several times, like after that Sunday, I thought: Do you know what? Now you have been wasting a whole day on nothing. Nothing, you have not done anything.”* (Participant 2)

Frustration with being fatigued was also described by the health professionals:

*“It takes time [to understand that they are fatigued] and it is a test of patience that is difficult for the patients to accept. They get really frustrated because they are so tired.”* (Focus group 3)

While having fatigue interferes with their emotions, the stroke survivors also described that experiencing irritation or sadness could also trigger or aggravate fatigue.

Having fatigue also interfered with stroke survivors’ *cognitive performance.* During periods of fatigue, the stroke survivors experienced difficulties in communicating, interacting socially, concentrating and maintaining attention. The stroke survivors further described that attempts to concentrate on a task, make decisions, and sustain attention towards a subject could often aggravate the fatigue. Health professionals reported that cognitively demanding rehabilitation activities, such as hand training, often resulted in patients getting excessively fatigued:

*“Previously a lot of patients performed physical activities regularly, but now they get tired in a completely different way. Performing upper limb rehabilitation … it is not a lot of repetitions before they get mentally exhausted, because they really need to concentrate and keep focusing.”* (Focus group 2)

PSF also interfered with the ability to perform regular *activities in daily life*, such as activities outside the home, household chores or sustained activities without rest, and even getting dressed or taking a shower in extreme instances. These activities in daily life were also reported to aggravate fatigue.

PSF interference with activities was further complicated by both the intensity and the diurnal variations of fatigue, leading to difficulties with anticipating their day-to-day capacity. This unpredictability was described as an essential and problematic consequence of having fatigue, and made life with PSF more challenging to manage:

*“And also, when you have something that you need to do … you don’t know when it [fatigue] will come or if you will get tired from things.”* (Participant 2)

The stroke survivors described that fatigue in general was an obstacle for them to *participate in society*. Stroke survivors of working age all reported that they worked less, or not at all, due to their fatigue. Being social, engaging in hobbies, and keeping in contact with friends and family were experienced as difficult due to fatigue.

*“It don’t have the same energy to be with them [my family] [ … ] Before, we used to babysit our grandchildren [ … ] but now, I don’t at all have the capacity to do the nice things anymore, and that’s too bad … ”* (Participant 7)

### Management

Both stroke survivors and health professionals described a continuous process of trying to find a balance and adapting to life with PSF. The stroke survivors gradually learned to recognize PSF characteristics, how PSF interfered with their life and its aggravating factors. The health professionals in this study described how they observed, advised and supported this process. Two different management strategies were identified: *emotional* and *behavioural strategies*.

Both stroke survivors and health professionals described different *emotional strategies.* Sometime after their stroke, the stroke survivors acknowledged that fatigue was part of their life and accepted that they had to adjust accordingly. Some also tried to ignore fatigue, and carried out activities despite knowing that participating would induce severe fatigue. The health professionals underlined the importance of patients needing to experience on their own how fatigue affected them, and that this was important in accepting the new situation:

*“They are used to having a lot of energy and suddenly they don’t. And I see that several patients need to go through everything a couple of times, where they get really fatigued, until they start prioritizing, and understand that this is how things need to be.”* (Focus group 2)

The stroke survivors described different *behavioural strategies* aimed at either preventing or relieving fatigue. Preventive strategies included limiting the activities performed, prioritizing, planning, structuring the days, resting in advance and seeking information.

*“It is a delicate balance between activities and rest. If I overdo it … sometimes I feel very good, and then I get on with training, but then mostly the next day and even the day after is ruined.”* (Participant 1)

The health professionals helped the patients to acknowledge their fatigue, advised them not to spend all their energy at once and helped them to find a balance between activities and rest:

“*I try to put on the breaks sometimes, because some of them are very eager [ … ] I advise them to take a break, this is a marathon, not a sprint.”* (Focus group 1)

To relieve fatigue, both stroke survivors and health professionals reported different strategies such as withdrawing from a situation, resting, and sleeping. For some stroke survivors, resting involved sitting still and solving a crossword puzzle (physical rest), some needed rest in a dark and quiet room devoid of stimulus (mental rest), whereas others described a combination of both these resting strategies.

### PSF awareness

Both stroke survivors and health professionals interpreted fatigue in the early phase as a normal reaction to being acutely ill. Later in the rehabilitation phase, depending on when they recovered from other stroke sequelae, the stroke survivors expected to return to their pre-stroke level of energy. However, when their fatigue did not resolve, their *awareness of PSF* became gradually evident. The health professionals reported that most stroke survivors were exhausted and tired in the early phase, but they did not define it as PSF, as fatigue during this stage was perceived as temporary:

*“We don’t call it fatigue in the acute phase, that’s more after a while when we can see how the damage manifests itself, because it is natural to be very tired and exhausted in the beginning.”* (Focus group 3)

Both stroke survivors and health professionals described PSF as evident when fatigue started to be in conflict with the patient’s and society’s expectations of performance, often occurring after their initial hospitalization due to their stroke.

*“When I came home from rehab … I was supposed to start doing things, inviting people and being social, things that I love to do. Then I did not have energy to do the things I did before. [ … ] we have this tradition inviting a lot of people. I was looking forward to it. But I was SO tired, and I did not understand it. I just sat there crying, I can’t do this, we have to call everybody and cancel.”* (Participant 5)

## Discussion

In this study exploring the PSF experiences of stroke survivors and PSF descriptions by health professionals, four themes were identified: PSF characteristics, interference and aggravating factors, management, and PSF awareness. PSF was described as an experience of mental, physical or general feeling of exhaustion and tiredness, with a discrepancy between the level of activity and the level of fatigue. PSF interfered with, and was aggravated by, emotions, cognitive performance, activities in daily life and participation in society. To manage PSF, both emotional and behavioral strategies were used. It took time before patients were aware of PSF, and it often became evident when fatigue resulted in inability to carry out expected daily activities.

### Themes and categories of PSF

The first theme, *characteristics of PSF*, contained five categories important for the characterization of PSF. In line with previous studies [18, 40], *quality* was described as mental, physical, and general fatigue. Likewise, the *diurnal variations* in fatigue intensity found in this study are in agreement with previous studies on PSF [18]. Further, intensity of and diurnal variations in PSF have also been found to be distinct from fatigue in other chronic conditions, such as multiple sclerosis [41]. Despite these prevalent findings, existing fatigue PROMs mostly lack items on quality subtypes and diurnal variations [9]. Interestingly, stroke survivors in this study experienced fatigue despite being in good physical condition, supporting a previous review that found no association between PSF and physical fitness [42]. In contrast, several existing fatigue PROMs include impaired physical condition as an indicator of fatigue. However, these fatigue PROMs are not developed or designed specifically to assess fatigue in a stroke population [9]. This emphasizes the problems of using a generic PROM to assess PSF and suggests that assessment of PSF requires a disease-specific PROM, which is currently not available.

Another theme of PSF in this study was *interfering and aggravating factors*. This included the categories: *emotions, cognitive performance, activities in daily life* and *participation in society*. Previous studies have shown that PSF leads to frustration and emotional disturbances [17], interferes with cognition [43], and also impacts activities at a social, family and community level [17, 19].

The third theme of PSF was *management*, including *emotional* and *behavioral strategies*. Accepting fatigue and adjusting expectations, applying energy-conservation strategies and resting both in advance of and after activity, as well as being physically active and receiving support from others have been previously described as strategies for managing PSF [16, 18, 19, 43]. Although limited evidence exists on the effectiveness of different management strategies, improved assessment and identification of such strategies will enable future studies to compare their effectiveness.

### Diagnostic criteria and PSF awareness

A major limitation of existing fatigue PROMs is the lack of clear diagnostic criteria. The stroke survivors in this study experienced fatigue in the early phase after stroke, similar to observations made by health professionals. Both stroke survivors and health professionals described how PSF interfered with the rehabilitation process. Nevertheless, both patients and health professionals interpreted PSF during this stage as a normal response, and did not necessarily recognize it as a significant problem. This is in agreement with a previous study reporting that fatigue first became evident during hospital admission, but the impact on role loss was not realized until after discharge [43]. In contrast, the case definition for PSF developed by Lynch et al. [8] contains individual criteria for detecting PSF during hospitalization, as the authors acknowledge PSF in the early phase to be important. In addition, a longitudinal observational study found that having PSF in the early phase after a stroke was an independent risk factor for poor physical health 18 months after stroke [3]. These prior studies highlight the importance of assessing and diagnosing PSF in the early phase after stroke.

As fatigue is a common symptom in the general population, not all fatigue experienced after a stroke should necessarily meet the definition of PSF. In individuals with a previous (pre-stroke) history of fatigue, PSF should only be considered when the feeling of fatigue is substantially different in its characteristics, and/or severely increased in intensity. For others, PSF should be considered when the feelings of fatigue are new and persistent after the stroke. However, to accurately distinguish newly developed post-stroke fatigue from pre-existing fatigue through the use of PROMs could be challenging. Including a retrospective item asking about pre-stroke fatigue history could be an important first step to investigating the potential similarities and differences between pre- and post-stroke fatigue.

### PSF as a multidimensional phenomenon

This study highlights the complexity and multidimensionality of PSF, which included closely interacting emotional, cognitive, physical and social aspects. When measuring complex constructs such as fatigue, a multidimensional measurement instrument is preferable in order to have a detailed assessment of all relevant dimensions [20]. For example, both symptom intensity and symptom interference measures are considered vital, as stroke survivors might report fatigue as very distressing and significantly interfering with daily life, despite reporting relatively low fatigue intensity, and vice versa. This is in agreement with symptom experience in cancer patients, showing a non-linear relationship between symptom severity and symptom interference [44]. In order to have a more comprehensive assessment of PSF that includes all relevant dimensions, there is a need for a new PSF-specific PROM.

### Study strengths and limitations

The study met all relevant COSMIN criteria, which are considered the gold standard for establishing content validity in PROM development [13]. Most of the COREQ criteria are also met, except returning transcripts and participant checking, as well as repeat interviews. We aimed to include a heterogeneous sample of participants in order to explore a broad range of experiences with PSF, but only nine stroke survivors participated in the study. In addition, stroke survivors were asked retrospectively about their fatigue experiences in the early phase and were interviewed up to 24 months post-stroke, introducing possible recall bias. Although the median age of the stroke survivors in this sample was low (59 years) compared to the median age for stroke in Norway (76 years) [45], our sample is too small for quantitative comparisons and the individual ages reflect an age distribution that is representative of the stroke populations in many countries. Another strength of this study was that the perspectives of health professionals working with stroke patients were also included, and results from these focus groups were largely consistent with results from the individual interviews with stroke survivors. Further, the overall aim of this study was to explore PSF to guide future development of a PSF-specific PROM. The results from this study will serve as the basis for item generation in the new PSF PROM. The drafted PROM will then be pilot-tested with cognitive interviews, giving the new participants the opportunity to add, modify and remove items.

## Conclusion

This study highlights the complexity and multidimensionality of PSF, which included closely interacting emotional, cognitive, physical and social aspects. Fatigue was interpreted as a normal reaction in the early phase after stroke, and awareness of PSF first emerged when PSF came into conflict with the patient’s and society’s expectations of performance. Since stroke survivors might not immediately recognize their fatigue, health professionals can help patients to comprehend and adapt to living with fatigue. The results of this study will form the basis for item generation and the development of a comprehensive PSF-specific PROM. Further studies will follow COSMIN-methodology for PROM development, which will include: drafting the PSF PROM, pilot-testing it with cognitive interviews, and field-testing the PROM in a larger sample to explore dimensions and potentially reduce items; further evaluation of the final PROM’s measurement properties will then be conducted in a cross-sectional sample [20].

## Supplementary Information

**Additional file 1 **: **Online resource 1**. Consolidated criteria for reporting qualitative studies (COREQ): 32-item checklist.

**Additional file 2 **: **Online resource 2**. Research questions and interview guides for individual interviews and focus groups.

## Data Availability

Not applicable.
