# Supplementary material for: Post-stroke fatigue: an exploratory study with patients and health professionals to develop a patient-reported outcome measure
Source: J Patient Rep Outcomes. 2021 Apr 21;5:35. doi: 10.1186/s41687-021-00307-z (PMC8060374; doi:10.1186/s41687-021-00307-z)
Supplement: Supplementary file 1 — Additional file 1 : Online resource 1. Consolidated criteria for reporting qualitative studies (COREQ): 32-item checklist. [file 41687_2021_307_MOESM1_ESM.pdf]

Online Resource 1. Consolidated criteria for reporting qualitative studies (COREQ): 32-item checklist

No.	Item	Response
**Domain 1: Research team and reflexivity**		
Personal characteristics		
1.	Interviewer/facilitator	The first author conducted all the interviews and the focus groups. One of the co-authors was a co-moderator in the first focus group and another co-author was a co-moderator in the last two focus groups.
2.	Credentials	The first author is a RN, MSc, and currently a PhD-student.
3.	Occupation	Nurse
4.	Gender	Female
5.	Experience and training	The first author has clinical experience as a nurse working with stroke patients. She conducted a pilot individual interview (not included in the data material due to >2 years post-stroke), in which transcripts were thoroughly reviewed by two co-authors in relation to interview skills. One of the co-authors is a highly experienced qualitative researcher, and have many years of research experience in this particular field. One of the co-moderators has previous experience with moderating focus groups and attended the first focus group.
Relationship with participants		
6.	Relationship established	The first author had no prior relationship with any of the stroke survivor participants, and had a prior working relationship with 6 of the health professionals in one of the focus groups.
7.	Participant knowledge of the interviewer	All participants were informed about the study aim prior to participation.
8.	Interviewer characteristics	As described above.
**Domain 2: study design**		
Theoretical framework		
9.	Methodological orientation and theory	Individual semi-structured interviews and focus groups.
Participant selection		
10.	Sampling	Purposive sampling method.
11.	Method of approach	*Individual interview participants:* We recruited participants from two different sites. First, we invited participants through a Facebook page for a stroke organization. A total of 25 potential participants reached out, and based on our purposive sampling strategy, five stroke survivors were selected to participate. Because the mean age of these participants was younger than the mean age of the general stroke population, we also chose to recruit participants from a stroke outpatient clinic at a local hospital. At this stage, we aimed to recruit participants over 60 years of age, in addition to diversity in other characteristics. We screened all patients over 60 years of age for fatigue at 3-month stroke follow up. We asked if they had fatigue and if it was a problem in their daily life. Four participants were invited to participate, and all consented to participation.

12. Sample size	A total of 9 stroke survivors and 16 health professionals participated in this study.
13. Non-participation	All individuals asked to participate in this study agreed, and there were no drop outs.
Setting	
14. Setting of data collection	Participants chose the interview location; five interviews were conducted in a hospital administration building, and four were conducted in the participants' homes. The health professionals participated in a focus group conducted at the participants' work place, during working hours.
15. Presence of non-participants	In one of the individual interviews, the participant's partner was at home in a nearby room, but no non-participants were present in the other interviews.
16. Description of sample	In two of the focus groups, all participants were acquainted, and in one of the focus groups there were participants from two different city districts.
Data collection	
17. Interview guide	The content of the interview guide was pilot-tested in an individual interview, and the final interview guide was adapted for both individual interviews and focus groups, as shown in Online Resource 2.
18. Repeat interviews	Repeat interviews were not performed.
19. Audio/visual recording	Interviews and focus groups were audio recorded.
20. Field notes	Yes.
21. Duration	Between 45-80 minutes
22. Data saturation	Reported in the article under "Data analysis".
23. Transcripts returned	Transcripts were not returned to participants.
**Domain 3: analysis and findings**	
Data analysis	
24. Number of data coders	The first author coded all the material and a co-author individually coded half of the material. The analysis was regularly discussed amongst the research team.
25. Description of the coding tree	Yes, please see Table 1 and "Data analysis".
26. Derivation of themes	Yes, please see "Results".
27. Software	NVIVO (v.11)
28. Participant checking	Participants did not provide feedback on the findings.
Reporting	
29. Quotations presented	Quotations are presented in the manuscript.
30. Data and findings consistent	Yes, documented in results.
31. Clarity of major themes	Please see Fig. 1 for the conceptual model describing
32. Clarity of minor themes	both major and minor themes
