# Supplementary material for: Post-stroke fatigue: an exploratory study with patients and health professionals to develop a patient-reported outcome measure
Source: J Patient Rep Outcomes. 2021 Apr 21;5:35. doi: 10.1186/s41687-021-00307-z (PMC8060374; doi:10.1186/s41687-021-00307-z)
Supplement: Supplementary file 2 — Additional file 2 : Online resource 2. Research questions and interview guides for individual interviews and focus groups. [file 41687_2021_307_MOESM2_ESM.pdf]

**Online Resource 2.** Research questions and interview guides for individual interviews and focus groups

**We applied the following research questions:**

1) How is PSF experienced/described from the perspectives of both stroke survivors and health professionals?
2) What is it like to live with PSF, from the perspectives of both stroke survivors (experienced) and health professionals (observed)?
3) How is PSF managed, both by stroke survivors and by health professionals?

**Interview guide – individual interviews**

- Did you experience fatigue prior to your stroke?
- If so, how would you describe your pre-stroke fatigue?
- Can you tell me about the first time you noticed being fatigued after your stroke?
- How did it affect your life at that time?
- How would you describe your fatigue right now?
- What makes you fatigued?
- How does having fatigue affect your life?
- How do you handle having fatigue?
- Are there any other aspects that are important in relation to this?

**Interview guide – focus groups**

- How do you define post-stroke fatigue?
- Can you tell me about some of your patients affected by PSF?
- How does PSF manifest?
- Can you describe factors that trigger fatigue?
- How does PSF affect patients' lives, including rehabilitation and use of health care services?
- How do you adapt your treatment to a patient with PSF?
- How can PSF be managed?
